# Identification of Actionable Mutations in Metastatic Castration-Resistant Prostate Cancer Through Circulating Tumor DNA: Are We There Yet?

**DOI:** 10.3390/curroncol32120692

**Published:** 2025-12-08

**Authors:** Wensi Tao, Amanda Sabel, R. Daniel Bonfil

**Affiliations:** 1 Department of Medical Education, Dr. Kiran C. Patel College of Allopathic Medicine, Nova Southeastern University, Fort Lauderdale, FL 33328, USA; wtao@nova.edu; 2 Dr. Kiran C. Patel College of Osteopathic Medicine, Nova Southeastern University, Fort Lauderdale, FL 33328, USA; as6252@mynsu.nova.edu

**Keywords:** ctDNA, liquid biopsy, biomarker, prostate cancer, mCRPC

## Abstract

Circulating tumor DNA (ctDNA) analysis has emerged as a powerful and minimally invasive approach for genomic profiling of metastatic castration-resistant prostate cancer (mCRPC), enabling real-time detection of tumor-derived mutations that guide therapy. Approximately 20% of mCRPC patients harbor alterations in homologous recombination repair (HRR) genes, most commonly *BRCA1/2* and *ATM*, which are actionable with different poly-(ADP-ribose) polymerase inhibitors (PARPIs) used as monotherapy or in combination with androgen receptor signaling inhibitors (ARSIs). A smaller subset of patients with mismatch repair deficiency (MMRd) or microsatellite instability-high (MSI-high) tumors may benefit from immune checkpoint blockade with pembrolizumab. Different FDA-approved liquid biopsy assays detect these actionable alterations when tissue biopsies are unavailable or insufficient. This review summarizes current evidence on ctDNA-based genotyping in mCRPC, highlighting clinically actionable mutations, corresponding targeted therapies, and technical and analytical considerations for clinical implementation. By capturing DNA shed from multiple metastatic sites, ctDNA profiling provides a comprehensive view of tumor heterogeneity and enables serial monitoring of molecular evolution. Overall, ctDNA analysis represents a transformative advance in precision oncology, supporting personalized treatment selection and ongoing assessment of therapeutic response in mCRPC.

## Simple Summary

Metastatic castration-resistant prostate cancer (mCRPC) refers to prostate cancer that has spread to distant organs and no longer responds to androgen deprivation therapy. Despite the availability of treatments that can alleviate symptoms and modestly extend survival, it remains an incurable disease at this stage. The analysis of prostate cancer cells can now identify specific gene changes—called actionable mutations—that guide the selection of targeted therapies more likely to be effective for individual patients. However, obtaining tumor biopsies is often invasive and technically challenging, particularly in patients with metastatic disease. Recently, simple blood tests that analyze circulating tumor DNA (ctDNA), fragments of DNA shed by cancer cells into the bloodstream, have emerged as much less invasive and more practical alternatives. This review underscores the current utility and significance of ctDNA in detecting actionable genomic mutations to inform treatment strategies for patients with mCRPC.

## 1. Introduction

Cell-free DNA (cfDNA), also known as circulating cell-free DNA, consists of short DNA fragments continuously released into bloodstream and other body fluids from apoptotic or necrotic cells [1]. In healthy individuals, most cfDNA derives from apoptotic nucleated cells and is typically nonmutant with a modal fragment length of ~166 bp, although contributions from necrosis and active secretion can also occur [1,2]. In cancer patients, shorter cfDNA fragments, typically ranging from 132 to 145 bp, known as circulating tumor DNA (ctDNA), are also present in the bloodstream [3]. This ctDNA serves as a form of liquid biopsy, and in various cancer types, its levels in plasma have been shown to positively correlate with tumor burden and disease progression [4].

ctDNA is currently being investigated for its diagnostic, prognostic, and predictive potential across various solid tumors [2]. Compared to traditional tissue biopsies, ctDNA detected in plasma provides a minimally invasive alternative capable of identifying and longitudinally monitoring tumor-specific genomic alterations [5]. Many of these alterations represent “actionable mutations”, which may be either somatic or germline, and can guide therapeutic decision-making, predict treatment response, and offer prognostic insight into disease progression [6].

In patients with prostate cancer (PC), a major clinical challenge arises when the disease progresses clinically, radiographically, or biochemically despite androgen deprivation therapy (ADT), leading to the development of castration-resistant prostate cancer (CRPC) [7]. Notably, 60–70% of patients progress to metastatic CRPC (mCRPC) within 5 years [8], a stage where current therapies have extended overall survival (OS) but have yet to offer a cure [9,10]. Next-generation sequencing (NGS) is now recommended for all PC patients with metastatic disease to guide targeted therapy and genetic risk assessment [11,12]. Furthermore, genomic testing has become an invaluable tool for stratification of mCRPC patients who are likely to benefit from recently approved targeted therapies, particularly those directed against tumors harboring actionable genomic mutations [13]. Despite its benefits, NGS analysis of tissue biopsies in mCRPC patients is invasive and often challenging to obtain from metastatic sites, particularly when confined to the skeleton, which is commonly affected in these patients [14]. Given these limitations, genomic profiling of ctDNA has emerged as a viable alternative. This approach offers several advantages, including its minimally invasive nature, the relatively high fraction of ctDNA within the cfDNA in patients with mCRPC, the ability to perform serial sampling for real-time molecular monitoring, and consistent evidence of high concordance in mutation detection between ctDNA and metastatic tissue [14,15,16]. Moreover, multiple studies have shown that ctDNA is detectable in a large proportion of mCRPC patients—typically in more than 85% of cases—although the exact rate varies depending on the study population, timing of sampling, and assay sensitivity [16,17].

In this narrative review, we examine actionable mutations in patients with mCRPC, summarize targeted therapies approved by the Food and Drug Administration (FDA) based on genomic alterations, evaluate the status of ctDNA-based genomic testing, and highlight key challenges, limitations, and future directions in this rapidly evolving field.

## 2. Actionable Genomic Mutations in mCRPC

Among the numerous genomic alterations identified in mCRPC, many contribute to disease progression but currently lack targeted therapies or do not inform clinical decision-making. These are referred to as “non-actionable mutations” and include, for example, frequent alterations in tumor suppressor genes such as *TP53* and *RB1*, which are associated with poor prognosis and treatment resistance but remain untargetable with existing therapeutic options [18]. In contrast, “actionable mutations” are genomic alterations that can inform clinical decision-making because they can either be directly targeted with approved therapies or create a dependency or vulnerability in another pathway than can be therapeutically exploited [19]. Understanding the distinction between these types of mutations is critical for guiding precision medicine strategies and improving clinical outcomes in mCRPC.

Androgen receptor (AR) splice variants, such as AR-V7, are clinically relevant biomarkers associated with resistance to AR-signaling inhibitors (ARSIs), including enzalutamide and abiraterone acetate, and may guide toward taxane-based chemotherapy [20]. However, it is important to note that these variants are not actionable mutations, as they result from alternative splicing at the mRNA level—rather than from DNA-level mutations—and currently lack FDA-approved targeted therapies.

Among the most well-established actionable alterations in mCRPC are loss-of function mutations in genes involved in homologous recombination repair (HRR), a critical process that ensures accurate repair of DNA double-strand breaks (DSBs), particularly during the S and G2 phases of the cell cycle in normal cells [21].

**Figure 1:**
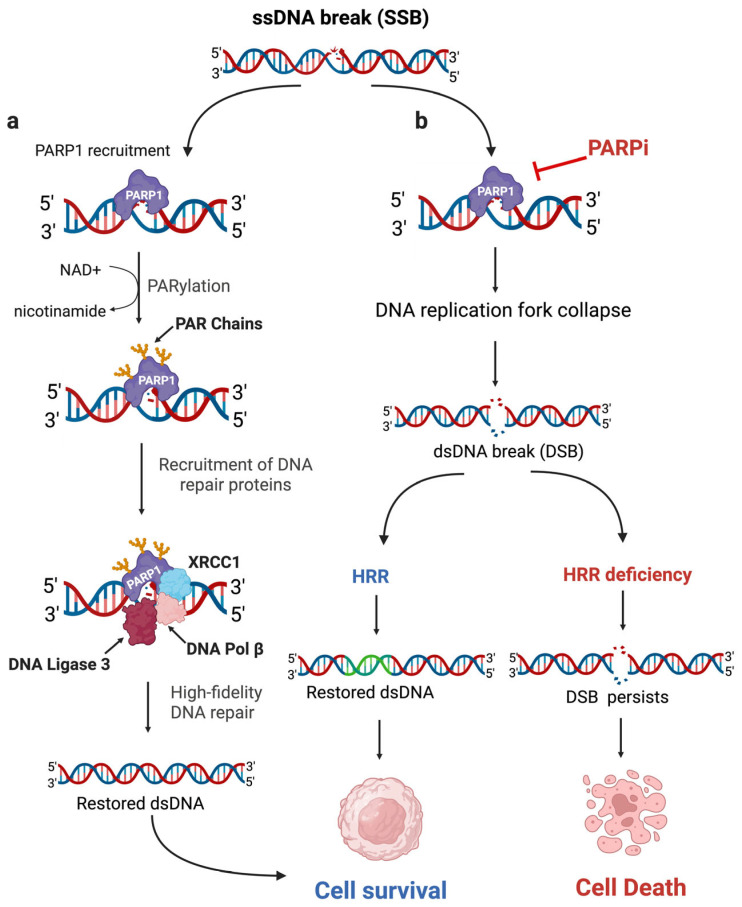
PARP1-mediated repair of single-strand DNA breaks in cells with differing HRR status. (**a**) PARP1 binds single-strand DNA (ssDNA) breaks and recruits repair proteins to restore DNA integrity. (**b**) PARPIs prevent repair, converting ssDNA breaks into double-strand DNA (dsDNA) breaks; HRR-proficient cells survive, while HRR-deficient cells accumulate dsDNA breaks and die. Created in BioRender. Tao, W. (2025) https://biorender.com/2ay3n8o (accessed on 7 December 2025).

To appreciate the significance of HRR dysfunction, it is helpful to first consider the broader context of DNA damage and repair. The most frequent DNA lesions are single-strand breaks (SSBs) [22], which are typically resolved by poly-(ADP-ribose) polymerase 1 (PARP1) [23]. Upon binding to an SSB, PARP1 catalyzes the transfer of ADP-ribose moieties from NAD^+^ to itself (auto-PARylation), generating long linear or branched poly-(ADP-ribose) (PAR) chains. This modification creates a docking platform for DNA repair proteins such as DNA ligase 3, XRCC1, and DNA polymerase β resulting in efficient repair of the damaged DNA [23] (Figure 1a). Failure to repair SSBs, such as upon PARP1 inactivation by PARP inhibitors (PARPIs), can convert them into DSBs when the replication fork encounters the lesion [24] (Figure 1b). Repairing these DSBs requires a functional HRR mechanism to ensure cell survival. In contrast, HRR deficiencies prevent proper repair, leaving DSBs unresolved and ultimately leading to cell death [25] (Figure 1b), caused by synthetic lethality. Both HRR and the concept of synthetic lethality are discussed below.

**Figure 2:**
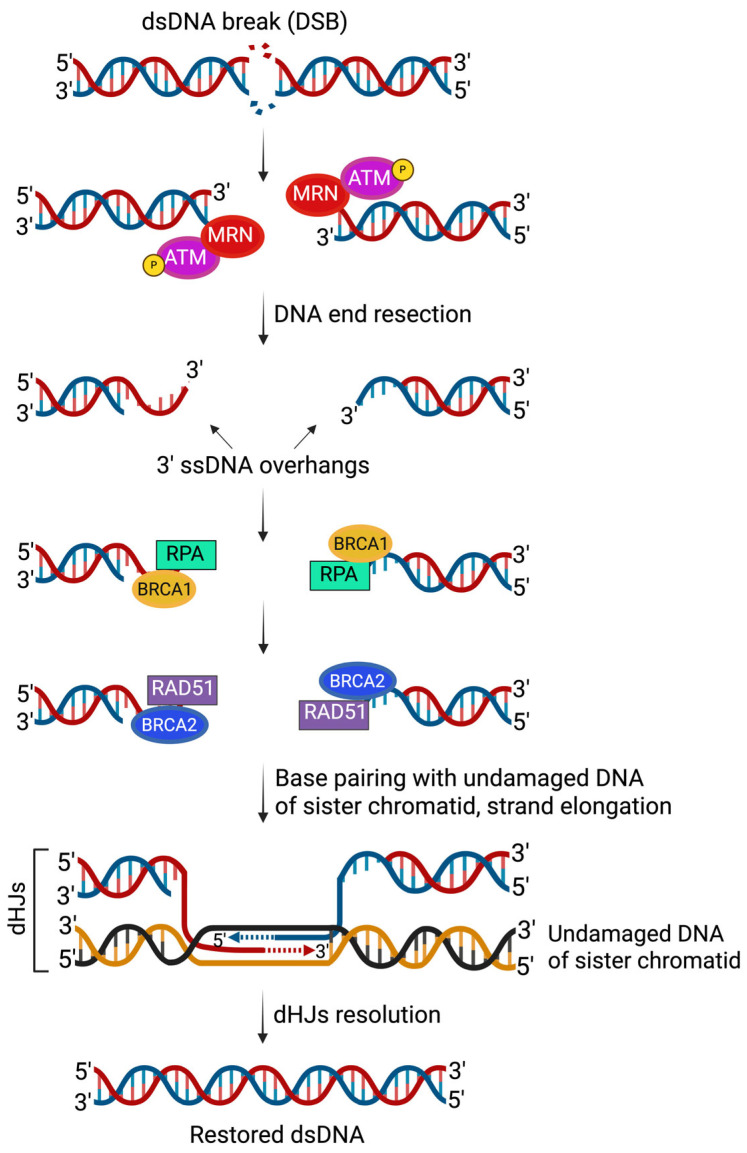
Simplified overview of homologous recombination repair (HRR). DSBs are processed by the MRN complex to generate 3′ ssDNA overhangs, which are coated by RPA. BRCA1/2 facilitate replacement of RPA with RAD51, forming a nucleoprotein filament that mediates homology search and strand invasion into the sister chromatid. Repair is completed through synthesis-dependent strand annealing or resolution of dHJs, restoring DNA integrity. Created in BioRender. Tao, W. (2025) https://biorender.com/2ay3n8o (accessed on 7 December 2025).

DSBs can be repaired through several pathways, among which the high-fidelity HRR mechanism is particularly important [24]. This process is initiated by the MRN protein complex (Mre11, Rad50, and Nbs1) which is recruited to the 5′ ends of DNA DSBs via Rad50 binding. In coordination with C-terminal-binding protein-interacting protein (CtIP), which activates Mre11’s endonuclease function, Mre11 introduces a nick in the 5′-terminated DNA strand and uses its 3′ → 5′ exonuclease activity to generate short 3′ single-stranded DNA (ssDNA) overhangs. Further resection is carried out primarily by the EXO1 exonuclease, generating long 3′ ssDNA overhangs that are essential for HRR. EXO1 activity is limited by ATM (ataxia-telangiectasia mutated), which avoids excessive DNA processing and helps recruitment of BRCA1. The exposed 3′ ssDNA overhangs are rapidly coated by replication protein A (RPA). Although BRCA1 does not physically interact with RPA, it facilitates the recruitment of BRCA2, which in turn mediates the replacement of RPA with RAD51 on ssDNA to form the nucleoprotein filament essential for HRR. The RAD51–ssDNA filament then searches for a homologous sequence on the sister chromatid, invades the intact duplex, and initiates DNA synthesis using the homologous strand as a template. Repair is completed either through synthesis-dependent strand annealing (SDSA) or resolution of a double Holliday junction (dHJ), both restoring DNA integrity with high fidelity [26,27,28,29]. A schematic representation that highlights key molecular events involved in HRR of DNA DSBs is shown in Figure 2.

Importantly, approximately 20% of patients with mCRPC harbor either somatic or germline mutations in HRR-related genes, including *ATM*, *BRCA1*, *BRCA2*, *NBN* (encoding Nbs1), and *RAD51D* [30,31]. Among these genes, *BRCA2* is the most frequently mutated, with somatic alterations being the predominant type [32].

Because *BRCA2*-deficient cells are unable to perform effective HRR, they become highly dependent on alternative repair pathways, particularly PARP-mediated repair [33]. This vulnerability has been therapeutically exploited through the concept of synthetic lethality, which occurs when defects in two genes, each non-lethal on its own, lead to cell death when combined (Figure 1b) [34]. This principle has been successfully translated into oncology, exemplified by the use of PARPIs to selectively target tumors with *BRCA1/2* mutations [35].

Another category of actionable alterations in mCRPC involves mutations in mismatch repair (MMR) genes, such as *MLH1*, *MSH2*, *MSH6*, and *PMS2* [36]. MMR deficiency (MMRd) leads to microsatellite instability-high (MSI-high) status, which increases the mutational burden of the tumor and its neoantigen load, thereby enhancing immune recognition [37].

## 3. Approved Targeted Therapies in mCRPC with Actionable Mutations

Different FDA-approved treatment modalities for mCRPC patients have recently been recommended by the National Comprehensive Cancer Network (NCCN) based on prior therapies administered and genetic testing [38]. Here, we will refer to therapy options for mCRPC patients, specifically for adenocarcinomas with actionable mutations in DNA repair pathways. Although nearly 20% of patients with mCRPC develop treatment-emergent small cell neuroendocrine prostate cancer (SCNC), mutations in DNA repair genes are reported to be mutually exclusive with SCNC tumors [39]. Therefore, therapeutic approaches for mCRPC with SCNC will not be described here.

Before initiating treatment in patients with mCRPC, it is crucial to perform HRR testing and review any prior therapies the patient may have received. For patients with HRR-mutated mCRPC who have progressed on treatment with ARSIs with no prior taxane-based therapy, FDA-approved regimens include PARPIs as monotherapy. For all patients with mCRPC, regardless of HRR mutation status, FDA-approved PARPIs can also be used in combination with ARSIs. PARPIs utilized include agents such as olaparib, rucaparib, talazoparib, and niraparib, while commonly used ARSIs include abiraterone acetate and enzalutamide.

Three clinical trials led to FDA approval of PARPIs as monotherapy in mCRPC patients. The PROfound trial (ClinicalTrials.gov identifier NCT02987543) established olaparib as the first PARPI approved for HRR-mutated mCRPC. In this study, patients with mCRPC who had progressed on prior ARSI therapy and carried at least one alteration in *BRCA1*, *BRCA2*, or *ATM* genes experienced significantly longer radiographic progression-free survival (rPFS) (median 7.4 vs. 3.6 months) and overall survival (OS) (median 19.1 vs. 14.7 months) with olaparib compared to those receiving standard ARSI treatment [40,41]. Post hoc analyses showed that the clinical benefits of olaparib were considerably less pronounced in patients with *ATM*-mutated tumors compared to those with *BRCA1* or *BRCA2* mutations [42].

Patients with mCRPC who had progressed on prior ARSI treatment, had not received previous chemotherapy, and harbored alterations in *BRCA1*, *BRCA2*, or *ATM* genes were also evaluated in the TRITON3 trial (ClinicalTrials.gov identifier NCT02975934), which instead investigated the PARPI rucaparib. In this trial, rPFS was significantly longer in the subgroup of patients harboring *BRCA1/BRCA2* mutations than in the physician’s choice of docetaxel or alternate ARSI control group (median, 11.2 vs. 6.4 months, respectively [43]. Although a slightly longer rPFS was found patients with mutations in *ATM* than in the control group (median, 8.1 vs. 6.8 months, respectively), the difference was not statistically significant [43]. *ATM* mutations are less frequent than *BRCA* mutations in mCRPC, accounting for 26% and 64% of cases, respectively, in the TRITON3 trial [43], which likely limited the statistical power for the *ATM* subgroup. As such, future clinical trials involving PARPIs may require larger patient cohorts to achieve sufficient statistical power to detect meaningful differences in treatment outcomes for rarer genetic alterations like *ATM* [44].

Based on the TRITON2 trial (ClinicalTrials.gov identifier NCT02975934), rucaparib has also been approved by the FDA for the treatment of patients with mCRPC harboring *BRCA1* or *BRCA2* mutations who progressed after one to two lines of ARSI therapy and one taxane-based chemotherapy [45]. Independent radiology review and investigator assessment confirmed objective response rates of 43.5% and 50.8%, respectively, which were similar among patients with either *BRCA1* or *BRCA2* mutations, whether germline or somatic.

In a multinational trial conducted across 17 countries, the PROpel trial evaluated the combination of olaparib and abiraterone as a first-line therapy for mCRPC patients without prior systemic treatment irrespective of their HRR gene mutation status. In the primary analysis, the combination significantly improved rPFS, with a median of 24.8 months versus 16.6 months in the placebo plus abiraterone arm, with a hazard ratio (HR) of 0.66 [46]. In the final prespecified analysis, the median OS was 42.1 months for the olaparib plus abiraterone arm compared to 34.7 months for the placebo plus abiraterone arm (HR 0.81), a difference that did not reach statistical significance [47]. However, this remains the longest reported median OS to date in a first-line mCRPC trial, reflecting a 19% reduction in the risk of death with the combination therapy. Importantly, in a *BRCA*-mutated subgroup within the study, patients treated with placebo plus abiraterone had a median rPFS of only 8 months, whereas rPFS was not reached with the combination therapy (HR 0.24). Based on these results, the FDA approved olaparib in combination with abiraterone (and prednisone or prednisolone) for mCRPC patients with identified or suspected *BRCA* mutation [48].

Another key study to assess combination therapies included the MAGNITUDE trial, which was specifically designed to evaluate the efficacy of niraparib in combination with abiraterone plus prednisone versus placebo plus abiraterone and prednisone as a first-line treatment for patients with mCRPC who had not received prior ARSI or chemotherapy in this setting, although such therapies could have been administered in earlier hormone-sensitive stages of disease. Unlike PROpel, the MAGNITUDE trial prospectively stratified patients with mCRPC based on HRR mutation status, enrolling both those with and without HRR gene alterations, with particular emphasis on *BRCA1* and *BRCA2* mutations. In the first interim analysis, the addition of niraparib significantly improved rPFS in the HRR-mutated group, particularly among patients with *BRCA1/2* alterations, while no advantage was observed in the HRR-nonmutated cohort [49]. In final analysis, treatment with niraparib and abiraterone also led to improved OS in mCRPC patients with HRR alterations, especially those with *BRCA1* or *BRCA2* mutations, after adjustment for baseline prognostic factors (median, 30.4 months with combination therapy vs. 28.6 months with placebo plus abiraterone; HR 0.79) [50].

Another pivotal phase III study evaluating PARPI-ARSI combinations was the TALAPRO-2 trial, which compared talazoparib plus enzalutamide versus placebo plus enzalutamide as first-line therapy for patients with mCRPC across 26 countries [51]. Similarly to MAGNITUDE, the trial stratified patients by HRR mutation status (HRR-deficient vs. non-deficient or unknown). Eligible patients could have received prior ARSI or chemotherapy in earlier hormone-sensitive stages but no systemic therapy in the castration-resistant setting. At the primary analysis, median rPFS for all mCRPC patients (with and without HRR alterations) treated with talazoparib plus enzalutamide was not reached, compared with 21.9 months for the placebo plus enzalutamide group (HR 0.63; *p* < 0.0001) [51]. In the final analysis, talazoparib plus enzalutamide significantly improved OS versus placebo plus enzalutamide (median 45.8 vs. 37.0 months; HR 0.80), with consistent benefit in the HRR-mutated cohort and a more modest effect in the HRR non-deficient or unknown group [52]. Notably, unlike MAGNITUDE, which showed benefit exclusively in HRR-mutated patients, and PROpel, which demonstrated rPFS and some OS improvements regardless of HRR status, TALAPRO-2 demonstrated a significant OS advantage in the overall intention-to-treat population, with the strongest effect observed in patients harboring HRR gene alterations, particularly *BRCA1/2* mutations. The three FDA-approved therapies based on the trials described above using PARPI-ARSI combinations are summarized in Table 1.

Although these trials collectively support the role of PARP inhibitors in mCRPC, differences in design affect how their findings should be interpreted clinically. PROfound and TRITON3 included patients with *BRCA1/2* or *ATM* mutations who had previously received ARSI therapy; however, neither trial was powered to evaluate *ATM*-mutated disease, resulting in strong benefit in *BRCA*-mutated tumors but only limited, often non-significant effects in *ATM* cohorts. MAGNITUDE and TALAPRO-2 prospectively stratified patients by HRR status, demonstrating clear benefit in *BRCA*-mutated tumors; MAGNITUDE showed no advantage in HRR–wild-type disease, while TALAPRO-2 demonstrated a broader OS benefit in the overall population. PROpel did not stratify by HRR status but also showed the greatest treatment effect in *BRCA*-mutated disease. Overall, PARP inhibitor benefit is most consistent in *BRCA1/2*-mutated mCRPC, while outcomes in *ATM*-mutated tumors remain modest, partly due to small sample sizes and biological differences, underscoring the need for trials powered to evaluate non-*BRCA* HRR subgroups.

Beyond HRR alterations, another molecularly defined subgroup of mCRPC (3 to 5% of cases) is characterized by MSI-high or MMRd, which has important therapeutic implications. In 2017, the U.S. FDA issued a landmark “tumor-agnostic” approval of pembrolizumab, an anti–PD-1 immune checkpoint inhibitor, for adult and pediatric patients with MSI-high or MMRd unresectable or metastatic solid tumors, regardless of the tumor’s origin [53]. This reflects the strong predictive value of these biomarkers for response to immune checkpoint inhibition and offers a meaningful treatment option for subset of mCRPC patients. In the phase 2 KEYNOTE-199 trial, pembrolizumab monotherapy showed limited overall benefit in unselected mCRPC patients, with only ~11% achieving a ≥50% decline in PSA, and ~3–4% revealing objective radiographic responses [54,55]. However, durable benefits occurred in patients with MSI-high or MMRd tumors. Building on this, the phase-2 PERSEUS1 trial (NCT03506997) evaluated pembrolizumab in mCRPC enriched for MMRd/MSI-high tumors and other molecular subtypes hypothesized to be immune-sensitive (e.g., high tumor mutational burden, CDK12 alterations). In this cohort, 7/25 patients (28.0%, 95% CI 12.1–49.4%) achieved a composite response by 24 weeks; notably, one patient with an MMRd tumor experienced a complete metabolic response by functional imaging that remained ongoing at ~49 months [56].

## 4. ctDNA Analysis for the Detection of Actionable Mutations in mCRPC

### 4.1. Analytical Validity

The FDA approvals of PARPIs for mCRPC have relied on detecting actionable mutations, such as those involving *BRCA1*, *BRCA2*, and *ATM* genes, through tumor tissue testing, which continues to be the gold standard [57]. This approach typically employs FDA-approved companion diagnostics, such as Foundation One^®^ CDx (Foundation Medicine, Inc., Boston, MA, USA), an NGS assay performed on DNA extracted from formalin-fixed paraffin-embedded (FFPE) tissue which identifies genomic alterations in 324 genes, including 14 HRR genes [58,59]. However, genomic profiling of tumor tissue in patients with mCRPC is often challenging due to limited sample size availability, suboptimal DNA quality or yield at extraction, inaccessibility of some visceral lesions, and the predominance of bone metastases, which are difficult to biopsy and process [57,60,61]. For instance, in the PROfound clinical trial, 31% of tumor tissue samples from mCRPC patients failed genomic profiling due to one or more of these limitations [40], highlighting the need for alternative testing approaches capable of reliably detecting actionable mutations, particularly when tumor tissue analysis is not feasible, to guide therapeutic decisions. In this context, a retrospective analysis of plasma-derived ctDNA from patients with mCRPC harboring *BRCA1*, *BRCA2*, and/or *ATM* mutations enrolled in the PROfound study (cohort A) showed that ctDNA testing effectively detected these alterations [60].

### 4.2. Clinical Validity

Several studies have demonstrated high concordance between ctDNA and matched tumor tissue in detecting clinically relevant alterations, including those in HRR genes [16,57,60,62], supporting ctDNA as a viable alternative when tissue samples are unavailable or inadequate. Moreover, retrospective analyses from PROfound showed that patients identified as harboring *BRCA1*/*BRCA2*/*ATM* alterations through ctDNA had clinical outcomes similar to those identified through tumor tissue, supporting the validity of ctDNA as a biomarker for treatment selection [60].

ctDNA has also demonstrated validity in identifying biomarkers for tumor-agnostic immunotherapy. Although MSI-high and MMRd tumors are uncommon in patients with mCRPC, their presence constitutes an actionable biomarker, as pembrolizumab is approved for the treatment of MSI-high, MMRd, or tumor mutational burden-high (TMB-H; ≥10 mutations per megabase) solid tumors, regardless of tumor origin [54]. MSI-high/MMRd/TMB-H status is typically determined through analysis of tumor tissue; however, in cases where tissue sampling is not feasible, plasma-derived ctDNA offers a validated alternative [63]. In an exploratory study, Foundation One^®^ Liquid CDx was used to analyze plasma-derived ctDNA from a cohort of 95 patients with mCRPC, identifying MSI-high status in two cases (2.1%). Both MSI-high cases carried mutations in *MSH2* or *MSH3* genes, and TMB-H status was observed exclusively in these samples. Although tumor tissue was not analyzed for MSI status—preventing assessment of concordance between ctDNA and tissue findings, these results support the potential utility of ctDNA analysis for identifying MSI-high/MMRd/TMB-H status in mCRPC patients [59]. In a retrospective multicenter case series, Guardant360^®^ CDx (Guardant Health, Redwood City, CA, USA)—a 74-gene FDA-approved companion diagnostic for solid tumors [63,64], was used to assess MSI status in plasma-derived ctDNA from mCRPC patients. Patients whose tumors were identified as MSI high by this assay demonstrated meaningful clinical benefit from pembrolizumab therapy, further supporting ctDNA analysis as a feasible method to select mCRPC patients for immune checkpoint inhibitor therapy when tissue testing is limited [65].

### 4.3. Clinical Utility

Under the current NCCN guidelines, plasma-derived ctDNA analysis is considered an acceptable testing option when a metastatic biopsy is unsafe or otherwise unfeasible, particularly if performed at the time of disease progression, as indicated by biochemical (PSA) or radiographic evidence [38]. In accordance with these recommendations, the U.S. FDA has approved FoundationOne^®^ Liquid CDx (Foundation Medicine, Inc., Boston, MA, USA) as a companion diagnostic for the detection of *BRCA1*, *BRCA2*, and/or *ATM* alterations in plasma-derived ctDNA from mCRPC patients to determine eligibility for PARPI therapy [66]. Unlike the tissue-based FoundationOne^®^ CDx that covers 324 genes, FoundationOne^®^ Liquid CDx is FDA-approved to detect and report substitutions, insertions and deletions (indels) in 311 genes [66].

### 4.4. Limitations

Despite its expanding role, ctDNA testing has recognized limitations. Shedding of ctDNA varies across patients and disease states, potentially reducing detection sensitivity in low-volume or bone-dominant metastases [57,60,61]. The NCCN guidelines recommend performing germline testing to identify pathogenic variants that might otherwise be misclassified as somatic alterations, potentially leading to misinterpretations in clinical decision-making [38]. The distinction between somatic and germline mutations is critical, resulting in vastly different consequences regarding treatment selection, prognosis, and family screening (Table 2).

Various tumor genomic sequencing platforms, including FoundationOne^®^ CDx and FoundationOne^®^ Liquid CDx, cannot reliably differentiate germline from somatic mutations [67]. This limitation highlights the importance of confirmatory germline testing when clinically indicated. In contrast, the BRACAnalysis CDx^®^ (Myriad Genetics, Salt Lake City, UT, USA) is an FDA-approved diagnostic specifically designed to detect germline *BRCA1* and *BRCA2* mutations using peripheral blood samples. This test is used to identify mCRPC patients eligible for PARPI therapy, enabling accurate identification of germline mutations essential for guiding targeted treatment decisions [68]. For immunotherapy biomarkers, current evidence remains based primarily on small case series and retrospective analyses rather than large prospective validation [65]. Additionally, assay performance characteristics, including gene coverage and sequencing depth, vary across platforms, which may influence the detection of low-frequency variants and interpretation of negative results. These constraints should be considered when integrating ctDNA into clinical decision-making for mCRPC.

Together, the assays described above represent the current FDA-approved companion diagnostics used to identify clinically actionable genomic alterations and determine patient eligibility for PARPI or PARPI/ARSI therapies in mCRPC. The gene alterations detected by these assays, along with biomarkers guiding tumor-agnostic pembrolizumab therapy, are summarized in Table 3.

## 5. Challenges and Limitations to ctDNA Analysis of Actionable Mutations in mCRPC

The use of ctDNA to identify actionable mutations in mCRPC offers significant advantages, primarily due to its minimally invasive nature. This feature allows serial monitoring of patients to track their response to treatment, detect the emerging resistance during therapy, and identify new alterations without the need for repeated, invasive tumor tissue biopsies [69,70]. Importantly, ctDNA analysis can provide a more comprehensive view of the tumor’s genetic landscape, as it reflects DNA shed from multiple metastatic lesions, capturing both inter- and intra-metastatic heterogeneity, often missed by single-lesion tissue biopsies [69,71]. Furthermore, higher ctDNA fractions (the proportion of ctDNA in total cfDNA) have been correlated with poorer clinical outcomes in patients with mCRPC, suggesting that ctDNA levels can provide valuable prognostic and predictive information [70,72].

Despite these benefits, the implementation of ctDNA testing in mCRPC remains limited, mainly confined to research settings or select academic centers [73]. Beyond these logistical barriers, plasma-derived ctDNA analysis in mCRPC faces important limitations. Although it is a clinically acceptable alternative for identifying actionable mutations when metastatic tissue biopsy is contraindicated or unfeasible, and several FDA-approved companion diagnostic assays are available, multiple variables can confound ctDNA genotyping, complicating its routine use.

A critical parameter influencing the accuracy of ctDNA profiling is the ctDNA fraction. When tumor DNA shedding is low, as might occur in patients with limited metastatic burden or in those with predominantly sclerotic metastases, the resulting low ctDNA fraction may reduce analytic sensitivity. Results that are negative for driver alterations can be distinguished based on ctDNA fraction levels. Low ctDNA fractions (<1%) can indicate an “indeterminate negative” result, as subsequent confirmatory tissue biopsy may still uncover a driver alteration [74]. For example, a patient with predominantly bone sclerotic metastases may show a negative plasma HRR panel despite harboring a true *BRCA1/2* or *ATM* mutation that is later identified on tissue sequencing. In such cases, true somatic alterations may fall below the limit of detection for current assays used for ctDNA analysis, leading to false-negative findings and missed identification of actionable variants, including *BRCA2* truncating mutations or homozygous deletions. Consequently, patients who could benefit from targeted treatments like PARPIs, either alone or in combination with ARSIs, may be inappropriately excluded in those cases [75,76]. In contrast, results that are negative with high ctDNA fractions (≥1%) indicate an “informative negative” result, providing confidence that no driver alterations are present in the tumor [74]. Although ctDNA fractions generally increase with disease burden [70], it remains essential to quantify ctDNA prior to genomic testing to ensure sufficient tumor-derived DNA is present for reliable results. This can be achieved through relatively low-cost assays. Recently, a machine-learning tool was developed to assess whether a patient’s ctDNA percentage is sufficient for informative genotyping in mCRPC, enhancing the reliability of liquid biopsy-based diagnostics [72].

Another important limitation of ctDNA genotyping is the potential for false-positive results caused by mutations originating from white blood cells, particularly those associated with clonal hematopoiesis of indeterminate potential (CHIP) [16,77]. This is particularly relevant in mCRPC patients, who are often older and therefore more likely to harbor CHIP. Notably, mutations in HRR genes used to guide PARPI therapy, such as *ATM*, appear at higher frequency in ctDNA than in tumor tissue in approximately 10% of cases due to CHIP [77], and may be misinterpreted as tumor-derived variants [16,77]. Although not yet standard in clinical practice, paired whole-blood genomic analysis offers a practical strategy to identify and subtract CHIP-derived mutations, thereby refining the distinction between tumor-specific and hematopoietic mutations. Incorporating such approaches into ctDNA workflows will be essential to improve the accuracy and reliability of molecular interpretation, particularly when ctDNA is used to guide therapeutic decisions such as PARP inhibitor eligibility.

In addition, relying solely on ctDNA analysis can sometimes lead to inaccurate interpretation of HRR gene status, potentially impacting therapeutic decision-making. Notably, except for BRACAnalysis CDx^®^, most FDA-approved ctDNA-based tumor genomic sequencing platforms do not distinguish between germline and somatic variants. Integrating concurrent sequencing of white blood cell DNA with ctDNA analysis allows for accurate variant classification, ensuring appropriate clinical management [73].

Finally, while tumor tissue TMB-H status serves as a predictive biomarker to identify mCRPC patients who may benefit from immune checkpoint inhibitors such as pembrolizumab, extrapolating TMB measurements from ctDNA requires caution. Plasma-based TMB estimates may not directly correspond to tissue-derived cutoff values used to predict treatment response, and their clinical utility in mCRPC remains to be fully validated [73].

## 6. Conclusions

The integration of ctDNA analysis into the clinical management of mCRPC represents a transformative advance toward precision oncology, enabling real-time, minimally invasive assessment of tumor evolution and treatment response. It is unequivocally ready to serve as the preferred alternative to tissue biopsy when metastatic tumor tissue is inaccessible, insufficient, or when serial monitoring is clinically indicated. In such contexts, ctDNA-based testing provides molecular insights that can directly inform therapeutic decisions and guide the use of targeted agents such as PARPI or immune checkpoint inhibitors.

**Figure 3:**
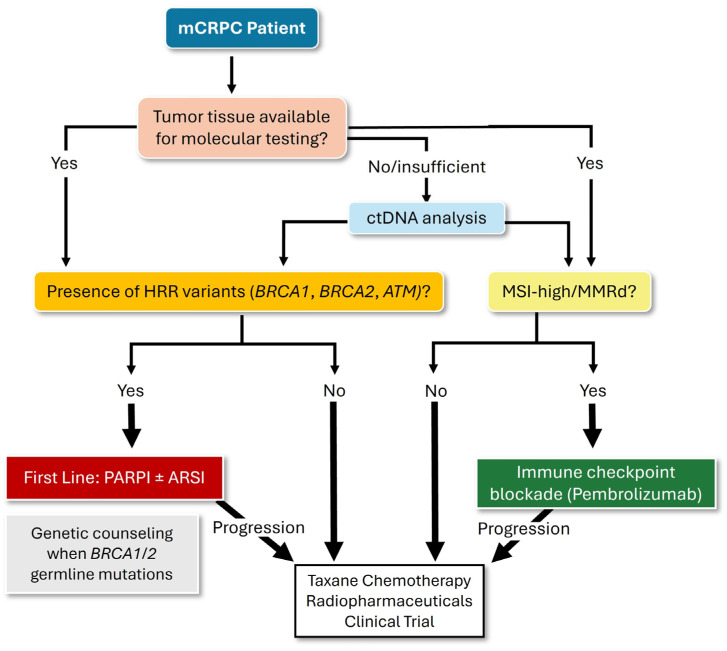
Treatment algorithm for mCRPC based on actionable genomic alterations. This flowchart illustrates a precision oncology framework for mCRPC integrating molecular profiling of tumor tissue or circulating tumor DNA (ctDNA) when tissue is insufficient or inaccessible. Detection of homologous recombination repair (HRR) gene alterations (e.g., *BRCA1*, *BRCA2*, *ATM*) supports the use of PARP inhibitors (PARPI), alone or in combination with androgen receptor signaling inhibitors, ARSI), with genetic counseling recommended for carriers of germline *BRCA1/2* mutations. Upon progression in mCRPC patients with HRR alterations, treatment sequencing involves chemotherapy, radiopharmaceuticals (e.g., ^177^Lu-PSMA-617 for patients with prostate-specific membrane antigen (PSMA)-positive imaging), or clinical trial, same as for cases with non-detectable HRR mutations. Tumors with mismatch repair deficiency (MMRd) or microsatellite instability–high (MSI-high) are eligible for immune checkpoint inhibition (pembrolizumab).

PARPI therapy (alone or in combination with ARSI) is the preferred first-line targeted option for patients with pathogenic HRR alterations detected in either tissue or ctDNA, with genetic counseling indicated for germline *BRCA1/2* carriers due to the associated hereditary cancer risk. Upon biochemical or radiographic progression, repeat ctDNA testing is essential—both to identify newly acquired resistance mechanisms (e.g., *BRCA* reversion mutations or *AR* alterations) and to detect previously missed HRR alterations. This iterative testing approach ensures that subsequent therapeutic decisions -including sequencing taxane-based chemotherapy, radiopharmaceuticals like ^177^Lu–PSMA-617 in patients with prostate-specific membrane antigen (PSMA)-positive disease, or clinical trial enrollment—remain aligned with the evolving molecular profile of the tumor. Patients with MSI-H/MMRd tumors -identified through tissue or ctDNA-are eligible for immune checkpoint blockade, whereas those lacking these alterations proceed with standard systemic therapies, similar to patients who have progressed on PARPI therapy or prior immunotherapy (Figure 3).

Despite its readiness as an alternative to tissue genotyping, ctDNA analysis is not yet suitable as a universal, standalone test that completely replaces tumor tissue profiling. Its reliability is constrained by the ctDNA fraction (with low shedding increasing false-negative risk), analytical sensitivity, and the need to distinguish tumor-derived mutations from CHIP or germline variants. Additionally, some platforms still lack the ability to reliably differentiate somatic from germline alterations.

Future progress will rely on standardization and optimization of analytical workflows, encompassing sequencing platforms and bioinformatic pipelines. Incorporating matched germline and white blood cell sequencing will enhance assay specificity, while prospective validation studies are necessary to confirm clinical concordance with tissue-based testing. Ultimately, ctDNA genotyping stands as a powerful complement and, in select scenarios, a practical substitute for tissue-based assays, but its optimal clinical utility depends on rigorous methodological consistency and evidence-based interpretation frameworks.

## Figures and Tables

**Table 1:** Summary of Clinical Trials Supporting FDA-approved Combination Therapies with PARPIs and ARSIs in mCRPC Patients.

Trial (NCT#)	Regimen	Population	Key Findings
PROpel (NCT03732820)	Olaparib + Abiraterone (vs. placebo + Abiraterone)	No prior systemic treatment. Irrespective of HRR status.	Overall rPFS benefit observed. No statistically significant OS benefit reached for the overall population, though the longest OS reported among first-line mCRPC trials to date. **HRR Mutations**: Strongest rPFS benefit observed, particularly with *BRCA* mutations. **No HRR Mutations**: Statistically significant rPFS benefit observed, though smaller than in HRR-positive group.
MAGNITUDE (NCT03748641)	Niraparib + Abiraterone (vs. placebo + Abiraterone)	No prior systemic treatment (except in earliest hormone-sensitive setting). HRR-mutated vs. HRR-wildtype	**HRR Mutations**: Significant rPFS improvement and longer OS, particularly with *BRCA* mutations. **No HRR Mutations:** No rPFS or OS benefit observed. The combination was stopped in this cohort due to statistical futility.
TALAPRO-2 (NCT03395197)	Talazoparib + Enzalutamide (vs. placebo + Enzalutamide)	No prior systemic treatment (except in earliest hormone-sensitive setting). HRR mutation not required but analyzed as subgroups.	Overall improvement in rPFS observed, with OS improvements observed regardless of HRR status. **HRR Mutations:** Strongest rPFS and OS improvements observed, particularly with *BRCA1/2* mutations. **No HRR Mutations:** Statistically significant rPFS improvement observed. OS benefit, though smaller than in HRR-positive group.

Abbreviations: PARPIs, Poly (ADP-ribose) polymerase (PARP) Inhibitors; NCT#, National Clinical Trial number; ARSIs, androgen receptor-signaling inhibitors; hazard ratio, HR; mCRPC, metastatic castration-resistant prostate cancer; rPFS, radiographic progression-free survival; OS, overall survival.

**Table 2:** Clinical and Familial Consequences of Somatic and Germline Variants in mCRPC.

Feature	Somatic Variant	Germline Variant
**Origin/Location**	Acquired; confined only to tumor cells.	Inherited; found in every cell of the body.
**Treatment Consequence**	Guides individual therapy (e.g., PARPI eligibility).	Guides individual therapy (e.g., PARPI eligibility) and may affect overall prognosis or choice of subsequent lines of treatment.
**Familial Consequence**	None: Cannot be passed to children or relatives.	Major. 50% risk of transmission to offspring, requiring genetic counseling and cascade testing for blood relatives.
**Patient Risk Consequence**	None regarding risk of other primary cancers.	High lifetime risk for other primary cancers (e.g., pancreatic), requiring enhanced screening.

**Table 3:** FDA-approved companion diagnostic used to identify HRR gene alterations determine eligibility for PARPI or PARPI/ARSI therapy in mCRPC patients, and molecular biomarkers guiding tumor-agnostic pembrolizumab therapy.

Assay	Sample Type	Coverage
FoundationOne^®^CDx	FFPE tissue	Alterations in 324 genes, including *BRCA1*, *BRCA2*, *ATM*, *BARD1*, *BRIP1*, *CDK12*, *CHEK1*, *CHEK2*, *FANCL*, *PALB2*, *RAD51B*, *RAD51C*, *RAD51D*, and *RAD54L* genes, as well as MSI-H, MMRd, and TMB-H.
FoundationOne^®^ Liquid CDx	Plasma cfDNA	Alterations in 311 genes—a more constrained version of FoundationOne^®^CDx.
Guardant360^®^ CDx	Plasma cfDNA	Alterations in >70 genes, including *BRCA1*, *BRCA2*, *ATM*, and MSI-high
BRACAnalysis CDx^®^	Peripheral (whole) blood	Germline mutations in *BRCA1* and *BRCA2* genes

Abbreviations: FDA, U.S. Food and Drug Administration; HRR, homologous recombination repair; PARPI, poly (ADP-ribose) polymerase (PARP) inhibitor; ARSI, androgen receptor-signaling inhibitor; mCRPC, metastatic castration-resistant prostate cancer; FFPE, formalin-fixed paraffin-embedded; MSI-high, microsatellite instability-high; MMRd, mismatch repair deficiency; TMB-H, tumor mutational burden-high; cfDNA, circulating tumor DNA.

## Data Availability

No new data were created or analyzed in this study.

## Abbreviations

The following abbreviations are used in this manuscript:

ADT	Androgen deprivation therapy
AR	Androgen receptor
ARSI	Androgen receptor-signaling inhibitor
ATM	Ataxia-telangiectasia mutated
Bps	Base pairs
cfDNA	Cell-free DNA/circulating cell-free DNA
CHIP	Clonal hematopoiesis of indeterminate potential
ctDNA	Circulating tumor DNA
CRPC	Castration-resistant prostate cancer
CtIP	C-terminal-binding protein interacting protein
dHJs	Double Holliday junctions
dMMR	Deficiency in mismatch repair
DSBs	DNA double-strand breaks
FDA	Food and Drug Administration
FFPE	Formalin-fixed, paraffin-embedded tissue
HRR	Homologous recombination repair
mCRPC	Metastatic castration-resistant prostate cancer
MMR	Mismatch repair
MMRd	Mismatch repair deficiency/deficient
MSI	Microsatellite instability
NCCN	National Comprehensive Cancer Network
NGS	Next-generation sequencing
OS	Overall survival
PARP1	Poly-(ADP-ribose) polymerase 1
PARPI	PARP inhibitor
PAR	Poly-(ADP-ribose)
PC	Prostate cancer
PFS	Progression-free survival
PSA	Prostate-specific antigen
PSMA	Prostate-specific membrane antigen
RPA	Replication protein A
rPFS	Radiographic progression-free survival
SCNC	Small-cell neuroendocrine prostate cancer
SDSA	Synthesis-dependent strand annealing
SSBs	Single-strand breaks
SsDNA	Single-stranded DNA
TMB	Tumor mutational burden
